# Decatecholaminisation during sepsis

**DOI:** 10.1186/s13054-016-1488-x

**Published:** 2016-10-04

**Authors:** Alain Rudiger, Mervyn Singer

**Affiliations:** 1 Institute of Anaesthesiology, University Hospital Zurich, Raemistrasse 100, CH 8091 Zurich, Switzerland; 2 Bloomsbury Institute of Intensive Care Medicine, University College London, London, WC1E 6BT UK

Sepsis is defined as life-threatening organ dysfunction caused by a dysregulated host response to infection [1]. The syndrome is characterised by autonomic dysfunction and increased plasma levels of noradrenaline and adrenaline [2]. These catecholamines originate mainly from the activated sympathetic nervous system, but also originate from the adrenal gland, gut, and immune cells [3]. While necessary and life-saving in the early fight or flight reaction to any insult, prolonged adrenergic stress is detrimental and contributes to organ dysfunction [4]. Strategies to reduce adrenergic stress have been proposed (Table 1) under the umbrella term decatecholaminisation.

**Table 1:** Decatecholaminisation strategies for patients with septic shock

	Strategy	Recommendations
Blunt endogenous catecholamine release; avoid compensatory adrenergic stimulation	Optimize cardiac preload and vascular filling	Assess fluid status by leg-raise test
		Perform repetitive fluid challenges to a target (e.g. stroke volume)
		Use cardiac output monitoring and/or echocardiography
	Treat hypoxia and severe anaemia	Target oxygen saturation between 92–96 %
		Transfuse red blood cells if haemoglobin falls below 70 g/l
	Optimize sedation and analgesia	Avoid over-sedation; use sedation targets
		Interrupt sedation daily, especially if long-lasting sedatives (e.g. midazolam) are used
		Use dexmedetomidine (see text for details)
Reduce exogenous catecholamine administration	Avoid excessive beta-mimetic stimulation	Use cardiac output monitoring and/or echocardiography Avoid supra-normal physiological targets
	Only use inotropes if contractility is impaired	Use cardiac output monitoring and/or echocardiography
	Consider alternative drugs	Consider alternative inotropes (e.g. levosimendan) and vasopressors (e.g. vasopressin)
	Accept abnormal physiological values	Adjust therapeutic targets
	Consider beta-blockers if tachycardia persists	Prefer short-acting drugs (e.g esmolol, see text) that can be stopped if adverse effects occur
Blunt inflammatory response (to reduce cardiac depression and microvascular dysfunction)	Treat underlying infection	Use intravenous antibiotics (after sampling for microbiology)
		Push for urgent surgical/interventional source control
	Reduce cytokine load	Consider low-dose steroids
		Consider extra-corporeal cytokine removal

Evidence and class of recommendations vary between the different interventions

Esmolol (Table 2) is a short-acting cardioselective beta-1 adrenergic blocker which has been tested in septic animals and in preliminary studies in human sepsis [5]. In the largest trial to date, Morelli et al. [6] enrolled septic shock patients with tachycardia (>95 beats/min) and an ongoing requirement for high-dose norepinephrine despite 24 h of active resuscitation. In this high-risk population (28-day mortality of 80.5 % in the control group), esmolol titrated to control heart rate was both safe and efficacious, reducing mortality to 49.4 %. The observed decrease in norepinephrine requirements could be mediated by a blunted immune response, resulting in an improved microcirculation [7], or enhanced adrenergic receptor sensitivity [8].

**Table 2:** Pharmacological properties of the study drugs

	Dexmedetomidine	Esmolol
Characteristics	Highly selective alpha-2 adrenoreceptor agonist	Short-acting, selective beta-1 blocker
Mode of action	Acts centrally, predominantly in the brain stem (sedation) and in the spinal cord (analgesia)	Acts peripherally, predominantly in the heart
Effects	Short- and long-term sedation in the intensive care unit setting	Negative chronotropic, dromotropic, inotropic effects
		Improves ventricular filling by prolonging diastole
	Anxiolysis; opioid-sparing effect; anti-delirant effects	Sympatholytic activity
	Sympatholytic activity	
Route of administration; dose	Intravenous infusion: 0.2–1.4 μg/kg/h Loading dose not recommended in clinical practice	Infusion: 25 mg/h, up-titration every 20 min in increments of 50 mg/h, to reach the target heart rate of <95beats/min
Pharmacokinetics	Half-life: 1.5 h	Half-life: 9 min
	Degradation by hepatic metabolism	Degradation by unspecific esterases
	No dose adjustments in renal dysfunction	No dose adjustment in renal and/or hepatic dysfunction
Adverse haemodynamic effects	Hypotension: 25 %, serious 1.7 %	Symptomatic hypotension: 12 %
	Hypertension: 15 %	Haemodynamic deterioration in patients with compensatory tachycardia
	Bradycardia: 13 %, serious 0.9 %	

Dexmedetomidine is a highly selective alpha-2 adrenoreceptor agonist that has sedative, anxiolytic, and opioid-sparing effects (Table 2) [9, 10]. The use of dexmedetomidine in critically ill patients increased ventilator-free time [11] and decreased the incidence of postoperative complications, delirium, and mortality up to 1 year post-cardiac surgery [12]. In postoperative patients, dexmedetomidine provided sympatholytic activity [13]. It also offers anti-inflammatory and organ protective effects in animal models [14]. The use of dexmedetomidine as an anti-adrenergic strategy in sepsis has been evaluated in a recently completed multicentre Japanese study (‘DESIRE’, https://clinicaltrials.gov/ct2/show/NCT01760967; last accessed 28 August 2016) for which results are still eagerly awaited.

In this issue of *Critical Care*, Hernandez et al. [15] tested both esmolol and dexmedetomidine in a sheep model of endotoxic shock with systemic hypotension, pulmonary hypertension, and hyperlactataemia. After a brief phase of fluid resuscitation and haemodynamic stabilisation with norepinephrine, animals were randomised to receive dexmedetomidine, esmolol, or placebo. Despite the early use of sympatholytic drugs, systemic and regional haemodynamics were maintained in the interventional groups compared to the control group over the 2-h study period. Although heart rate was significantly reduced by esmolol, cardiac output, mean arterial pressure, noradrenaline requirements, and SvO_2_ did not differ from placebo-treated animals. Dexmedetomidine reduced serum adrenaline levels by almost 40 %. Both esmolol and dexmedetomidine reduced arterial and portal vein lactate levels and improved lactate clearance. In summary, both drugs were well tolerated from a haemodynamic point of view and associated with likely beneficial effects on metabolism.

These observations are particularly interesting as dexmedetomidine and esmolol were started very early after shock induction. However, the short duration of the study precludes knowledge of longer term effects and any impact on outcomes. Furthermore, it would have been fascinating to have a fourth experimental group exploring possible synergism between esmolol and dexmedetomidine, as a rationale could be argued for the use of both. Certainly it is premature to translate these findings to clinical practice in septic patients, but this work should encourage further research into the role of alpha-2 agonists in sepsis, with or without beta-blockade.

## References

[1] Singer M, Deutschman CS, Seymour CW, Shankar-Hari M, Annane D, Bauer M, Bellomo R, Bernard GR, Chiche JD, Coopersmith CM (2016). The Third International Consensus Definitions for Sepsis and Septic Shock (Sepsis-3). JAMA..

[2] Annane D, Trabold F, Sharshar T, Jarrin I, Blanc AS, Raphael J-C, Gajdos P (1999). Inappropriate sympathetic activation at onset of septic shock: a spectral analysis approach. Am J Respir Crit Care Med..

[3] Rudiger A, Singer M (2013). The heart in sepsis: from basic mechanisms to clinical management. Curr Vasc Pharmacol..

[4] Andreis DT, Singer M. Catecholamines for inflammatory shock: a Jekyll-and-Hyde conundrum. Intensive Care Med. 2016;42:1387-97. 10.1007/s00134-016-4249-z 26873833

[5] Rudiger A (2010). Beta-block the septic heart. Crit Care Med..

[6] Morelli A, Ertmer C, Westphal M, et al. Effect of heart rate control with esmolol on hemodynamic and clinical outcomes in patients with septic shock: a randomized clinical trial. JAMA. 2013;310:1683–91. 10.1001/jama.2013.278477 24108526

[7] Morelli A, Donati A, Ertmer C, Rehberg S, Kampmeier T, Orecchioni A, D’Egidio A, Cecchini V, Landoni G, Pietropaoli P (2013). Microvascular effects of heart rate control with esmolol in patients with septic shock: a pilot study. Crit Care Med..

[8] Reeves RA, Boer WH, DeLeve L, Leenen FH (1984). Nonselective beta-blockade enhances pressor responsiveness to epinephrine, norepinephrine, and angiotensin II in normal man. Clin Pharmacol Ther..

[9] Keating GM (2015). Dexmedetomidine: a review of its use for sedation in the intensive care setting. Drugs..

[10] Cruickshank M, Henderson L, MacLennan G, Fraser C, Campbell M, Blackwood B, Gordon A, Brazzelli M (2016). Alpha-2 agonists for sedation of mechanically ventilated adults in intensive care units: a systematic review. Health Technol Assess..

[11] Reade MC, Eastwood GM, Bellomo R, Bailey M, Bersten A, Cheung B, Davies A, Delaney A, Ghosh A, van Haren F (2016). Effect of dexmedetomidine added to standard care on ventilator-free time in patients with agitated delirium: a randomized clinical trial. JAMA..

[12] Ji F, Li Z, Nguyen H, Young N, Shi P, Fleming N, Liu H (2013). Perioperative dexmedetomidine improves outcomes of cardiac surgery. Circulation..

[13] Talke P, Richardson CA, Scheinin M, Fisher DM (1997). Postoperative pharmacokinetics and sympatholytic effects of dexmedetomidine. Anesth Analg..

[14] Hofer S, Steppan J, Wagner T, Funke B, Lichtenstern C, Martin E, Graf BM, Bierhaus A, Weigand MA (2009). Central sympatholytics prolong survival in experimental sepsis. Crit Care..

[15] Hernandez G, Tapia P, Alegria L, et al. Effects of dexmedetomidine and esmolol on systemic hemodynamics and exogenous lactate clearance in early experimental septic shock. Crit Care. 2016;20:234. 10.1186/s13054-016-1419-x PMC4969982 27480413

